# The Function of the NMDA Receptor in Hypoxic-Ischemic Encephalopathy

**DOI:** 10.3389/fnins.2020.567665

**Published:** 2020-10-06

**Authors:** Xiaotong Zhang, Kaizhen Peng, Xiaomin Zhang

**Affiliations:** School of Basic Medicine, Kunming Medical University, Kunming, China

**Keywords:** NMDA receptor, hypoxic-ischemic encephalopathy, GluN2A, GluN2B, excitotoxicity

## Abstract

Hypoxic-ischemic encephalopathy (HIE) is one of the main forms of neonatal brain injury which could lead to neonatal disability or even cause neonatal death. Therefore, HIE strongly affects the health of newborns and brings heavy burden to the family and society. It has been well studied that N-methyl-D-aspartate (NMDA) receptors are involved in the excitotoxicity induced by hypoxia ischemia in adult brain. Recently, it has been shown that the NMDA receptor also plays important roles in HIE. In the present review, we made a summary of the molecular mechanism of NMDA receptor in the pathological process of HIE, focusing on the distinct role of GluN2A- and GluN2B-containing NMDA receptor subtypes and aiming to provide some insights into the clinical treatment and drug development of HIE.

## Introduction

The clinical definition of hypoxic-ischemic encephalopathy (HIE) is “asphyxia of the umbilical blood supply to the human fetus occurring at 36 gestational weeks or later” (Millar et al., 2017). This is one of the most common causes of long-term neuronal impairment in children (Perlman, 1997, 2006; Maxwell et al., 2020). Studies have shown that the incidence rate of HIE for full-term newborns with more than 36 weeks gestational age is 3/1,000 (Knox et al., 2013; Hagberg et al., 2015), and this is approximately 7/1,000 for premature fetus within the gestational age of 33–35 weeks (Chalak et al., 2012). When the gestational age is less than 32 weeks, the rate significantly increases (Oskoui et al., 2013). In 2013, Oskoui et al. reported an incident rate of 62/1,000 for 28–31 weeks fetuses and more than 146/1,000 for less than 28 week fetuses (Oskoui et al., 2013).

Perinatal hypoxic-ischemic injury is characterized by high incidence and high mortality. It can cause permanent injury to the newborn and even endanger life. The newborns who survive usually suffer from various neurological disabilities, including developmental delay, cerebral palsy, epilepsy, visual impairment, and learning disabilities (Li et al., 2017; Koehler et al., 2018). In developed countries, the incidence of hypoxic-ischemic brain damage per thousand neonates is approximately 1.5 cases (Glass, 2018), while in developing countries, the incidence increases to 26 cases per thousand neonates (Li et al., 2017).

## Pathological Process of Neonatal Hypoxic-Ischemic Encephalopathy

The pathological process of neonatal HIE includes energy failure, oxidative stress, inflammation, and excitotoxicity. Together, these mechanisms lead to neuronal apoptosis, swelling, and necrosis. Under normal physiological conditions, the human brain has a high demand for oxygen and glucose, which are used to produce adenosine triphosphate (ATP) (Rocha-Ferreira and Hristova, 2016). In early HIE, due to the reduced supply of oxygen and blood flow, brain tissue turns to depend on anaerobic respiration for ATP, which will result in the depletion of ATP and decreased level of oxidative phosphorylation, followed by a series of abnormal cellular activities, such as severe metabolic acidosis induced by lactic acid accumulation. This was primary energy failure that occurred within 6 h after HIE (Juul and Ferriero, 2014). The destruction of cell homeostasis can lead to the imbalance of Na^+^, Ca^2+^, and water, as well as excitatory neurotransmitter release, resulting in “excitatory oxidative cascade reaction” (Juul and Ferriero, 2014). Then secondary energy failure occurs after 6 h, resulting in mitochondrial dysfunction and failure of energy metabolism in mitochondria. This would continue to cause excitotoxicity, oxidative stress, and inflammation and aggravate the brain tissue damage (Yang and Lai, 2011; Rocha-Ferreira and Hristova, 2015). These serious reactions promote the initial inflammatory reaction (Juul and Ferriero, 2014) and further cause secondary neuronal damage, which may last for several days, followed by the anti-inflammatory stage and repair stage (Juul and Ferriero, 2014; Rocha-Ferreira and Hristova, 2015). Furthermore, studies have shown the activation of glia cells, including the microglia, astrocytes, and oligodendrocytes in neonatal hypoxic-ischemic brain damage, activates immune response and results in the release of a large number of proinflammatory cytokines that promote neuronal apoptosis (Li et al., 2017; Mamun et al., 2020).

There are some differences between neonatal and adult hypoxic ischemia. For example, in early development, the N-methyl-D-aspartate (NMDA) receptor-mediated selective vulnerability increased (Huang and Castillo, 2008). Compared with adults, the developing brain is high in plasticity and also more susceptible to external stimuli (Rocha-Ferreira and Hristova, 2016). For example, it is shown that HIP3 (suffered to HI injury at postnatal day 3) rat tolerates better than HIP11 (suffered to HI injury at postnatal day 11) rat because of its more resilient metabolism (Odorcyk et al., 2020), but the sustained changes of brain metabolism may in turn influence the long-term cognitive development (Azevedo et al., 2020). Furthermore, although the immature brain can exhibit a certain resistance when it is subjected to hypoxia alone (Johnston et al., 2001), the coexistence of hypoxia and ischemia could reduce the resistance of neonatal brain, exaggerate neuronal excitability, and thereby cause neurons to more likely be damaged, and the injury would develop for several days (Johnston et al., 2001). In addition, the immature brain can endure long-term energy consumption due to its low energy demand. However, when the exhaustion of energy reaches a threshold, this would eventually activate the excitatory pathways and aggravate the damage (Rocha-Ferreira and Hristova, 2016). Moreover, it is recently reported that HIE could also impact brain development by interfering with the maturation of several types of cells in the central nervous system. For example, the maturation of oligodendrocytes is impaired after HIE and therefore causes abnormal myelination (Ziemka-Nalecz et al., 2018; Baldassarro et al., 2020; Janowska et al., 2020). Another study found that HIE impairs GABAergic development in hippocampus which might be the cause of HIE-induced long-term memory deficiency (Chavez-Valdez et al., 2020).

## The NMDA Receptor Is Involved in Neonatal Hypoxic-Ischemic Encephalopathy

Glutamate mediates most of the excitatory transmissions in the central nervous system. There are three types of ionergic glutamate receptors in the mammalian brain: NMDA receptor, AMPA receptor, and KA receptor (Yang and Lai, 2011). The NMDA receptor has high affinity for glutamate (Rosenberg and Aizenman, 1989; Rosenberg et al., 1992; Káradóttir et al., 2005). Therefore, it plays an important role in glutamate-mediated excitotoxicity (Rosenberg and Aizenman, 1989; Rosenberg et al., 1992; Lynch and Guttmann, 2002; Káradóttir et al., 2005; Makarewicz et al., 2014) and is also involved in many physiological processes (Forrest et al., 1994). The physiological activity of the NMDA receptor is essential for normal neurological function, including synaptic plasticity, cognition, learning, and memory formation. However, the excessive release of glutamate under pathological conditions leads to the over-activation of NMDA receptors, resulting in excessive Ca^2+^ influx that activates downstream death signaling pathways and finally leads to cell necrosis or apoptosis, which is known as excitotoxicity (Szydlowska and Tymianski, 2010; Lai et al., 2011; Zhou et al., 2013; Wu et al., 2017; Wu and Tymianski, 2018). NMDA receptors exist in neurons and glial cells of newborns (Jantzie et al., 2015) and are composed of four different subunits, including the structural subunit GluN1 and the regulatory subunits GluN2 and GluN3 (Traynelis et al., 2010; Paoletti et al., 2013). GluN1 knockout in mice is lethal in the perinatal period, indicating its important role in development and survival (Zhou et al., 2013). GluN2 subunits are regulatory subunits, which determine the biophysical and pharmacological properties of different NMDA receptor subtypes and affect the assembly, downstream signaling, transport, and synaptic targeting of NMDA receptors (Lau and Zukin, 2007). GluN2 subunits have four different types, namely, GluN2A, GluN2B, GluN2C, and GluN2D. Among these, GluN2A and GluN2B are the major subunits expressed in frontal cortex and hippocampus (Monyer et al., 1994). At the early stage of development, the GluN2B-containing NMDA receptor is dominant, and the expression of the GluN2A-containing NMDA receptor gradually increases in the later stage of development and eventually outnumbers the GluN2B-containing NMDA receptor (Knox et al., 2013). Studies have shown that neonatal hypoxic-ischemic injury is correlated to NMDA receptor-mediated excitotoxicity (Johnston et al., 2002; Wu et al., 2017). Furthermore, it has also been proven that after 6 h of hypoxia ischemia, the expression of GluN2A significantly decreases, while the expression of GluN2B exhibits an opposite trend, which reaches a maximum at 24 h after hypoxic ischemia (Lai et al., 2016). This suggests that GluN2A and GluN2B might play different roles in neonatal HIE.

### The Role of the GluN2B-Containing NMDA Receptor in Hypoxic Ischemic Injury

Membrane-associated guanylate kinase (MAGUK) is a neuronal scaffold protein that interacts with GluN2A and GluN2B (Niethammer et al., 1996). The two MAGUK family members, postsynaptic density protein 95 (PSD95) and synapse-associated protein 102 (SAP102), were found to interact with GluN2A and GluN2B in adult brains (Lim et al., 2002). In neonatal neurons, the GluN2B/SAP102 complex is dominant, while the GluN2A/PSD95 complex gradually increases during development (van Zundert et al., 2004). In the early stage after HIE, the interaction between GluN2B and MAGUK decreases (Shao et al., 2017; Lu et al., 2018), while PSD95 subsequently increases its binding with GluN2B (Shao et al., 2017).

Neonatal hypoxia ischemia can rapidly activate the phosphorylation network of GluN2B and regulate the function of the NMDA receptor by recruiting new proteins, including Src family kinase (SFK), protein kinase C (PKC), calmodulin kinase (CaMKII), and other kinases, to the postsynaptic dense region (PSD) (Shao et al., 2017). SFK plays the role of the molecular center in NMDA receptors coupled signaling cascade (Jiang et al., 2008). After neonatal hypoxic-ischemic injury, SFK is activated in the PSD area (Jiang et al., 2011) and interacts with the GluN2B subunits (Knox et al., 2013). Fyn as a member of SFK mainly mediates tyrosine phosphorylation at three sites of GluN2B, including tyrosine (Y) 1472, Y1336, and Y1252 (Nakazawa et al., 2001). It has been shown that after neonatal HIE, the phosphorylation of Y1472 would increase (Knox et al., 2014). Furthermore, the overexpression of Fyn enhances the phosphorylation of GluN2B in neonatal HIE (Jiang et al., 2008) and exaggerates the neuronal damage, suggesting the pro-death role of SFK in GluN2B-mediated neuronal death. It has also been reported that in early stage of HIE, the phosphorylation of DAPK1 and the P85 subunit of PI3K (Lu et al., 2018) increases. These kinases are known to phosphorylate GluN2B and amplify its downstream pro-death signals (Takagi et al., 2003; Tu et al., 2010). Hence, they might also play similar roles in HIE. Although most researches have indicated the pro-death role of GluN2B in HIE, there is also evidence that GluN2B could protect neurons against apoptosis during development (Martel et al., 2009; Lai et al., 2016). Since the GluN2B-containing NMDA receptor is the dominant subtype in early development, it remains to be determined whether GluN2B plays dual roles in HIE.

### The Role of the GluN2A-Containing NMDA Receptor in Hypoxic Ischemic Injury

The expression of GluN2A in neonatal brain is relatively low in neonatal brains, and it was found that the expression of GluN2A further decreases after neonatal HIE (Lai et al., 2016). It has been suggested that excessive GluN2A phosphorylation in neonates increases the NMDA receptor-mediated excitotoxicity and affects normal brain function (Gurd et al., 2002). Therefore, the downregulation of GluN2A after 6 h of hypoxia ischemia injury might play a protective role (Lai et al., 2016). As mentioned above, after HIE, the SFK is activated and recruited into PSD (Shao et al., 2017). In addition to binding with GluN2B, they also interact with GluN2A (Gurd et al., 2002; Jiang et al., 2011). However, although tyrosine phosphorylation of GluN2A was detected in adult rats (Hardingham, 2009), no change was detected in neonatal rats (Takagi et al., 2002). Another possible explanation is that HIE inhibits the developmental switch of subtypes of the NMDA receptor from the GluN2B dominant to GluN2A dominant, and this might cause long-term cognitive impairment. Overall, despite the extensive studies of GluN2A in adult hypoxic ischemia, its role in neonatal HIE is not clear at present and requires further investigation.

### The NMDA Receptor Subtype Hypothesis and Neonatal Hypoxic-Ischemic Encephalopathy

Activation of NMDA receptor can induce neuronal survival or death during central hypoxia ischemia injury, and there are different hypotheses about the mechanism of this dual function of the NMDA receptor. The most famous hypotheses include location hypothesis and subtype hypothesis (Wu and Tymianski, 2018).

The location hypothesis suggests that the synaptic NMDA receptors are coupled to pro-survival signaling pathways, while the extrasynaptic NMDA receptors triggers neuronal death (Wu and Tymianski, 2018). For example, during ischemia, synaptic NMDA receptors activate the phosphoinositide 3 kinase (PI3K)-protein kinase B (PKB, also known as Akt) pathway and extracellular signal-regulated kinase (ERK)-cAMP response element-binding protein(CREB) pathway (Hetman and Kharebava, 2006; Zhang et al., 2007; Hardingham, 2009; Zhou et al., 2013) and exerts protective function. When synaptic NMDA receptor is opened, PI3K is phosphorylated and activated by calmodulin (Joyal et al., 1997) and further phosphorylates Akt (Impey et al., 2002), followed by a series of downstream survival cascades. In addition, synaptic NMDA receptors can also induce survival gene expression through activating Ras-ERK pathway and phosphorylating CREB (Wu et al., 2001; Hardingham et al., 2002; Impey et al., 2002; Hardingham, 2009). In contrast to the synaptic NMDA receptor’s role in protection, the extrasynaptic NMDA receptor is coupled to the death signaling pathway. The activation of extrasynaptic NMDA receptors deactivates the CREB pathway (Hardingham et al., 2002; Vanhoutte and Bading, 2003; Lai et al., 2011), and at the same time, it also phosphorylates ERK pathway to prevent CREB activation, promote the expression of death-promoting genes, and cause neuronal death (Vanhoutte and Bading, 2003; Hardingham, 2009). Compared to adult neurons, a mature spine structure is rare in neonate brain. Therefore, it remains unclear whether the location hypothesis of NMDA receptor also stands in HIE. Another possibility is that binding with different signaling cascades that are designated to be synaptic and extrasynaptic in mature neuron determines the role of GluN2A and GluN2B in HIE.

The subtype hypothesis suggests that different NMDA receptor subtypes mediate different downstream signaling molecules and play different roles in ischemia (Lai et al., 2011; Wu et al., 2017). In adult brains, the GluN2B-containing NMDA receptor is enriched outside the synapses and coupled to pro-death signaling cascade, while the GluN2A-containing NMDA receptor is mainly expressed in the synapse and mediates neuroprotection and neuronal survival (Liu et al., 2007; Chen et al., 2008). The roles of different NMDA receptor subtypes in hypoxia ischemia have been extensively studied, but a consensus is still not reached. Studies have shown that the activation of GluN2B-containing NMDA receptors is more lethal than GluN2A-containing NMDA receptors (Liu et al., 2007; Choo et al., 2012; Zhou et al., 2013; Sun et al., 2015). This suggests that GluN2B is the main hub of NMDA receptor-mediated excitotoxicity and plays a leading role in inducing cell death (Wu et al., 2017). However, some other researches show an opposite role of the GluN2B-containing NMDA receptor (Soriano et al., 2008; Martel et al., 2009).

Researches on the roles of GluN2B in neonatal HIE are relatively abundant than GluN2A, which is perhaps due to its high expression. The existing studies indicated that GluN2B might have both pro-death and protective roles in HIE. Although the activation of the pro-death signaling network coupled to GluN2B is activated early after HIE (Jiang et al., 2008; Knox et al., 2014; Lu et al., 2018), it has been proven that the GluN2B-containing NMDA receptors may also promote neurogenesis in the subventricular zone (Lai et al., 2016). Compared to GluN2B, the role of GluN2A is even more unclear. The expression of GluN2A is extremely low in early development and is further decreased after HIE (Lai et al., 2016), which makes it difficult to contribute much in mediating protection in neonate brain. However, because the developmental switch of GluN2A and GluN2B is required for the normal cognition, the lasting low level of GluN2A may interfere with the normal development of numerous neuronal functions.

Overall, since the expression pattern of GluN2A and GluN2B of newborn is quite different from that of adult, it is possible that their role in neonatal HIE may be different from adults.

### NMDA Receptors Participate in Glia-Mediated Neuronal Damage and Protection

Under normal physiological conditions, microglia in neonatal brain are more active than in adults (Li et al., 2017), these participate in synaptic pruning and neurogenesis (Matcovitch-Natan et al., 2016). The activation of microglia can be detected after HIE (Hagberg et al., 2015; Zhao et al., 2016), along with increase of glutamate, reactive oxygen species (ROS), and nitric oxide (NO), which cause oxidative damage and promote secondary energy failure (Jellema et al., 2013; Kaur et al., 2013). Astrocytes play an important role in maintaining the integrity of the blood–brain barrier and are responsible for glutamate transport (Anderson and Swanson, 2000). They regulate the homeostasis of extracellular ions, such as sodium and calcium ions (Lian and Stringer, 2004; Li et al., 2017). In addition, activated astrocytes secrete various chemokines to attract immune cells to migrate to the injured area and further aggravate hypoxic-ischemic brain damage (Miller et al., 2005; Koh et al., 2015; Zhao et al., 2016). In adult ischemic brain, astrocytes play roles in both pro-inflammation and anti-inflammation (Dong and Benveniste, 2001). However, the function of astrocytes in neonatal HIE remains unclear. It has been speculated that in neonatal HIE, astrocytes may function to attenuate inflammation (Li et al., 2017). The activation of glutamate receptors in oligodendrocytes leads to massive calcium influx and accumulation in mitochondria, contributing to the production of oxygen free radicals and caspase family-mediated cell death (Sánchez-Gómez et al., 2003; Matute et al., 2006). There is evidence that oligodendrocytes and its precursor cells express NMDA receptors that comprise GluN1 and GluN2A, GluN2B, GluN2C, or GluN3A, in which the most abundant were GluN2C- and GluN3A-containing NMDA receptors (Káradóttir et al., 2005; Koodziejczyk et al., 2009). It was also shown that the NMDA receptor participates in the myelination of oligodendrocytes in neonatal rats (Káradóttir et al., 2005), and during ischemia, the activation of NMDA receptors results in the Ca^2+^-dependent detachment and disintegration of oligodendroglial processes (Káradóttir et al., 2005; Salter and Fern, 2005), which might contribute to developmental delay caused by HIE.

## Treatment of Neonatal Hypoxic-Ischemic Encephalopathy

At present, there are few ways to effectively treat neonatal hypoxic-ischemic injury. Among these, hypothermia is the most widely used (Wassink et al., 2019). Hypothermia can intervene some critical steps in the excitatory oxidative cascade. It can reduce energy failure and protect the blood–brain barrier and thereby reduce the glutamate release and nitric oxide production and alleviate neuronal death (Thoresen et al., 1995; Kim et al., 2011; Wassink et al., 2014; Sun et al., 2019). And it is recently reported that HIE can also upregulate the expression and releasing of erythropoietin (EPO) in astrocyte to inhibit neuronal apoptosis (Toriuchi et al., 2020). In addition, hypothermia can also improve the survival of hypoxic-ischemic injury and benefit the development of the nervous system in moderate and severe HIE (Tagin et al., 2012). In the clinic, hypothermia is the standard treatment for hypoxic ischemic injury in neonates. After the birth of the child, the body temperature is reduced to 33.5°C immediately for 72 h. However, excessively low temperatures or long durations may lead to more severe damage (Martinello et al., 2017). Although hypothermia can provide some protection against hypoxic ischemic injury, improve neurocognition, and reduce mortality (Jacobs et al., 2011; Tagin et al., 2012), statistics show that merely one sixth of the patients benefit from this treatment (Dixon et al., 2015). Thus, in order to provide a complete neuroprotective effect, other adjuvant therapies are required (Adstamongkonkul and Hess, 2017).

Drugs such as melatonin, edaravone, erythropoietin (EPO), and growth factor have also been used for the treatment of hypoxic ischemic injury (Hua et al., 2017; Zhou et al., 2020). Melatonin has antioxidant, anti-inflammatory, and anti-apoptotic effects (Alonso-Alconada et al., 2013). In animal models, melatonin combined with hypothermia therapy has protective effect, which shows a good prospect as an adjuvant therapy (Merchant et al., 2013; Shea and Palanisamy, 2015; Cardinali, 2019). However, further research is needed on the dosage and dosing time window (Dixon et al., 2015). Edaravone is an antioxidant that can remove hydroxyl radicals, peroxyl radicals, and superoxide free radicals. It can reduce blood–brain barrier dysfunction and reduce infarct size (Lapchak, 2010). A clinical study revealed that edaravone can improve memory and learning ability after several days of hypoxic-ischemic injury (Lee et al., 2007). EPO can resist cell apoptosis, reduce excitotoxicity, and promote the repair after injury by regulating neuronal differentiation. In addition, EPO can also promote post-damage repair by regulating the differentiation of neurons (Lee et al., 2007; Zhu et al., 2009; Wu et al., 2012). However, although clinical trial studies have shown that EPO combined with hypothermia therapy is safe and effective (Hua et al., 2017; Nonomura et al., 2019), a recent study revealed that the therapeutic function of EPO may overlap with hypothermia, since these two treatments share intracellular signaling cascades (Wassink et al., 2020).

Since NMDA receptor-mediated excitotoxicity is the main cause of HIE pathological damage, inhibiting NMDA receptors to reduce excitotoxicity has also been considered a potential treatment for HIE. At present, studies have explored the therapeutic effects of magnesium, noble gas xenon, and a variety of NMDA receptor antagonists in HIE.

Magnesium sulfate can exert neuroprotective effects by inhibiting the activation of excitatory neurotransmitters (such as glutamate) of NMDA receptors, but the effects of magnesium on perinatal hypoxic-ischemic injury are not very uniform. Prenatal administration of magnesium sulfate has been shown to have neuroprotective effects on premature newborns (Doyle et al., 2007; Juul and Ferriero, 2014; Cho et al., 2015; Solevåg et al., 2019). However, in the hypoxic-ischemic piglet and neonate rat model, magnesium sulfate failed to effectively reduce severe tissue damage (Penrice et al., 1997; Zhu et al., 2004). In addition, the use of magnesium sulfate did not improve the neuron loss in the fetal sheep model with umbilical cord occlusion (Groenendaal et al., 2002). Although the combined treatment of hypothermia and MgSO_4_ has a certain effect in reducing the risk of death and improving short-term adverse consequences, it appears to be only effective for some children (Galinsky et al., 2014). Hence, further evaluations are needed to determine whether this is effective for the long-term survival and neurodevelopment of children (Rahman et al., 2015; Nonomura et al., 2019).

The noble gas xenon is shown to have neuroprotective effects in hypoxic-ischemic models through inhibiting NMDA receptors by competing for the binding of glycine in many *in vitro* and *in vivo* animal models (Hobbs et al., 2008; Zhuang et al., 2010; Faulkner et al., 2011; Zhao et al., 2013; Juul and Ferriero, 2014; Alam et al., 2017; Rüegger et al., 2018; Koziakova et al., 2019). Xenon can also be used as an adjuvant with hypothermia therapy to treat neonatal HIE and has been proven to be effective in reducing brain injury and improving long-term recovery (Ma et al., 2005; Chakkarapani et al., 2010; Amer and Oorschot, 2018). However, at present, there is not enough evidence to support xenon as a conventional clinical adjuvant neuroprotective agent (Rüegger et al., 2017; Amer and Oorschot, 2018). Hence, further studies are required to optimize its application for human neonatal hypoxia ischemia.

Recent studies have shown that the non-competitive NMDA receptor antagonist memantine has neuroprotective effects on hypoxic-ischemic brain injury *in vivo* (Landucci et al., 2018), but the damage or protection of memantine is correlated to the dose. Melissa Trotman et al. (2015) reported that low-dose memantine treatment can significantly reduce infarct volume and improve behavioral prognosis, while higher doses of memantine can significantly aggravate injury. The study conducted by Solevåg et al. (2019) revealed that memantine combined with low temperature can produce greater neuroprotective effects (Liu et al., 2020). There is still controversy on the effect of another non-competitive antagonist, MK801. Most studies have considered that in neonatal hypoxic-ischemic injury, MK801 alone or in combination with hypothermia can exert a neuroprotective effect, and its effect is enhanced when applied together with hypothermia (McDonald et al., 1987; Olney et al., 1989; Ikonomidou et al., 1999; Alkan et al., 2001; Gerriets et al., 2003). However, another study revealed that although MK801 can reduce necrotic cell death, it can activate caspase-3 in cortical GABAergic interneurons, thereby aggravating the apoptosis (Desfeux et al., 2010). In addition, studies have shown that MK801 may cause other side effects, including inhibiting the spontaneous activity of mice, seizure, or increase mortality (Ikonomidou et al., 1999; Liu et al., 2020), suggesting that MK801 may have a dual effect or that its effect is correlated to the type of neuron.

In addition, a variety of inhibitors of NMDA receptors have been shown to have protective effects in adult hypoxic ischemia. For example, ifenprodil and Tat-NR2B9c have neuroprotective effects in adult ischemic brain injury (Cui et al., 2007; Chen et al., 2008; Sun et al., 2008; Amico-Ruvio et al., 2012; Bhatt et al., 2013). Among these, ifenprodil can reduce ischemic cell death and enhance the neuroprotection induced by preconditioning (Chen et al., 2008), Tat-NR2B9c interferes with the interaction between NMDA receptors and PSD95 to protect neurons against excitotoxicity and reduce ischemic damage (Cui et al., 2007; Bach et al., 2012; Liu et al., 2020). However, it remains unclear whether these antagonists also play a protective role in neonatal ischemic brain injury, because the role of NMDA receptors and their intracellular signaling are different between neonates and adults.

Importantly, it was found that the application of NMDA receptor antagonist in neonates may cause abnormal neurodegeneration (Ikonomidou et al., 1999; Olney et al., 2002; Jevtovic-Todorovic et al., 2003; Nikizad et al., 2007), because the activation of the NMDA receptor is required for the normal development of the brain (Adesnik et al., 2008). Therefore, the safety and long-term effect of applying NMDA receptor antagonist for HIE treatment requires further evaluation. Furthermore, present protective agents in adult ischemia based on NMDA receptor, ifenprodil and Tat-NR2B9c, exert their protective function through inhibiting the GluN2B-containing NMDA receptor, which is the major type of NMDA receptor in neonate brains (Sheng et al., 1994; Cull-Candy et al., 2001; Liu et al., 2004). Therefore, normal function of NMDA receptors may be more substantially inhibited by these two agents in neonates than in adults. More comprehensive studies are needed to address this issue.

## Conclusion

Hypoxic ischemic injury in newborns is correlated to NMDA receptor-mediated excitotoxicity. After HIE, the over-activation of NMDA receptor leads to excessive Ca^2+^ influx and results in cell damage (Monyer et al., 1994). Compared with adults, neonatal brains are more susceptible to excitotoxic damage (Gurd et al., 2002), while the main mechanism may be the overexcitability of NMDA receptors (Monyer et al., 1994). The study of hypoxia ischemia in adult rodents revealed that GluN2A may mediate the survival effect through the ERK-CREB pathway (Terasaki et al., 2010), while GluN2B may play a lethal role through the GluN2B-PSD95-nNOS pathway (Wu et al., 2017). However, for neonates, since the expression of GluN2A and GluN2B is different from that of adults, the role of different NMDA receptors in mediating survival and lethal signaling pathways may be different. Recent studies have indicated that GluN2B-containing NMDA receptors may exaggerate damage but promote neurogenesis in the subventricular zone of neonatal rats (Nakazawa et al., 2001; Jiang et al., 2008; Lai et al., 2016). Therefore, regarding the important role of NMDA receptor in neuronal development, as well as the difference between NMDA receptor subtype expression, trafficking, and function between neonate and adult brains, further studies are needed to fully investigate the specific mechanism of NMDA receptors in neonatal hypoxic-ischemic injury and develop new drugs, ways, and methods to treat neonatal hypoxic ischemia.

## Author Contributions

XMZ: conceptualization. XMZ and XTZ: writing—original draft preparation. KZP, XTZ, and XMZ: writing—review and editing. XMZ: funding acquisition. All authors contributed to the article and approved the submitted version.

## Conflict of Interest

The authors declare that the research was conducted in the absence of any commercial or financial relationships that could be construed as a potential conflict of interest.

## References

[B1] Adesnik H. Li G. During M. J. Pleasure S. J. Nicoll R. A. (2008). NMDA receptors inhibit synapse unsilencing during brain development. *Proc. Natl. Acad. Sci. U.S.A.* 105 5597–5602. 10.1073/pnas.0800946105 18375768 PMC2291097

[B2] Adstamongkonkul D. Hess D. C. (2017). Ischemic conditioning and neonatal hypoxic ischemic encephalopathy: a literature review. *Cond. Med.* 1 9–16. 30215057 PMC6131706

[B3] Alam A. Chun Suen K. Hana Z. Sanders R. D. Maze M. Ma D. (2017). Neuroprotection and neurotoxicity in the developing brain: an update on the effects of dexmedetomidine and xenon. *Neurotoxicol. Teratol.* 60 102–116. 10.1016/j.ntt.2017.01.001 28065636 PMC12850681

[B4] Alkan T. Kahveci N. Buyukuysal L. Korfali E. Ozluk K. (2001). Neuroprotective effects of MK 801 and hypothermia used alone and in combination in hypoxic-ischemic brain injury in neonatal rats. *Arch. Physiol. Biochem.* 109 135–144. 10.1076/apab.109.2.135.4271 11780774

[B5] Alonso-Alconada D. Álvarez A. Arteaga O. Martínez-Ibargüen A. Hilario E. (2013). Neuroprotective effect of melatonin: a novel therapy against perinatal hypoxia-ischemia. *Int. J. Mol. Sci.* 14 9379–9395. 10.3390/ijms14059379 23629670 PMC3676788

[B6] Amer A. R. Oorschot D. E. (2018). Xenon combined with hypothermia in perinatal hypoxic-ischemic encephalopathy: a noble gas, a noble mission. *Pediatr. Neurol.* 84 5–10. 10.1016/j.pediatrneurol.2018.02.009 29887039

[B7] Amico-Ruvio S. A. Paganelli M. A. Myers J. M. Popescu G. K. (2012). Ifenprodil effects on GluN2B-containing glutamate receptors. *Mol. Pharmacol.* 82 1074–1081. 10.1124/mol.112.078998 22936815 PMC3502619

[B8] Anderson C. M. Swanson R. A. (2000). Astrocyte glutamate transport: review of properties, regulation, and physiological functions. *Glia* 32 1–14. 10975906

[B9] Azevedo P. N. Zanirati G. Venturin G. T. Schu G. G. Durán-Carabali L. E. Odorcyk F. K. (2020). Long-term changes in metabolic brain network drive memory impairments in rats following neonatal hypoxia-ischemia. *Neurobiol. Learn. Mem.* 171:107207. 10.1016/j.nlm.2020.107207 32147586

[B10] Bach A. Clausen B. H. Møller M. Vestergaard B. Chi C. N. Round A. (2012). A high-affinity, dimeric inhibitor of PSD-95 bivalently interacts with PDZ1-2 and protects against ischemic brain damage. *Proc. Natl. Acad. Sci. U.S.A.* 109 3317–3322. 10.1073/pnas.1113761109 22343531 PMC3295328

[B11] Baldassarro V. A. Marchesini A. Giardino L. Calzà L. (2020). Differential effects of glucose deprivation on the survival of fetal versus adult neural stem cells-derived oligodendrocyte precursor cells. *Glia.* 68 898–917. 10.1002/glia.23750 31755592

[B12] Bhatt J. M. Prakash A. Suryavanshi P. S. Dravid S. M. (2013). Effect of ifenprodil on GluN1/GluN2B N-Methyl-D-aspartate receptor gating. *Mol. Pharmacol.* 83 9–21. 10.1124/mol.112.080952 23007555

[B13] Cardinali D. P. (2019). An assessment of melatonin’s therapeutic value in the hypoxic-ischemic encephalopathy of the newborn. *Front. Synapt. Neurosci.* 11:34. 10.3389/fnsyn.2019.00034 31920617 PMC6914689

[B14] Chakkarapani E. Dingley J. Liu X. Hoque N. Aquilina K. Porter H. (2010). Xenon enhances hypothermic neuroprotection in asphyxiated newborn pigs. *Ann. Neurol.* 68 330–341. 10.1002/ana.22016 20658563

[B15] Chalak L. F. Rollins N. Morriss M. C. Brion L. P. Heyne R. Sánchez P. J. (2012). Perinatal acidosis and hypoxic-ischemic encephalopathy in preterm infants of 33 to 35 weeks’ gestation. *J. Pediatr.* 160 388–394. 10.1016/j.jpeds.2011.09.001 22033298 PMC3740949

[B16] Chavez-Valdez R. Lechner C. Emerson P. Northington F. J. Martin L. J. (2020). Accumulation of PSA-NCAM marks nascent neurodegeneration in the dorsal hippocampus after neonatal hypoxic-ischemic brain injury in mice. *J. Cereb. Blood Flow Metab.* 2020:271678X20942707. 10.1177/0271678X20942707 32703109 PMC8054724

[B17] Chen M. Lu T. J. Chen X. J. Zhou Y. Chen Q. Feng X. Y. (2008). Differential roles of NMDA receptor subtypes in ischemic neuronal cell death and ischemic tolerance. *Stroke* 39 3042–3048. 10.1161/STROKEAHA.108.521898 18688011

[B18] Cho G. J. Hong H. R. Hong S. C. Oh M. J. Kim H. J. (2015). The neuroprotective effect of magnesium sulfate in preterm fetal mice. *J. Perinat. Med.* 43 537–543. 10.1515/jpm-2014-0176 25503462

[B19] Choo A. M. Geddes-Klein D. M. Hockenberry A. Scarsella D. Mesfin M. N. Singh P. (2012). NR2A and NR2B subunits differentially mediate MAP kinase signaling and mitochondrial morphology following excitotoxic insult. *Neurochem. Int.* 60 506–516. 10.1016/j.neuint.2012.02.007 22366650 PMC4946952

[B20] Cui H. Hayashi A. Sun H. S. Belmares M. P. Cobey C. Phan T. (2007). PDZ protein interactions underlying NMDA receptor-mediated excitotoxicity and neuroprotection by PSD-95 inhibitors. *J. Neurosci.* 27 9901–9915. 10.1523/JNEUROSCI.1464-07.2007 17855605 PMC6672641

[B21] Cull-Candy S. Brickley S. Farrant M. (2001). NMDA receptor subunits: Diversity, development and disease. *Curr. Opin. Neurobiol.* 11 327–335. 10.1016/S0959-4388(00)00215-4 11399431

[B22] Desfeux A. El Ghazi F. Jégou S. Legros H. Marret S. Laudenbach V. (2010). Dual effect of glutamate on GABAergic interneuron survival during cerebral cortex development in mice neonates. *Cereb. Cortex* 20 1092–1108. 10.1093/cercor/bhp181 19759125

[B23] Dixon B. J. Reis C. Ho W. M. Tang J. Zhang J. H. (2015). Neuroprotective strategies after neonatal hypoxic ischemic encephalopathy. *Int. J. Mol. Sci.* 16 22368–22401. 10.3390/ijms160922368 26389893 PMC4613313

[B24] Dong Y. Benveniste E. N. (2001). Immune function of astrocytes. *Glia* 36 180–190. 10.1002/glia.1107 11596126

[B25] Doyle L. W. Crowther C. A. Middleton P. Marret S. (2007). Magnesium sulphate for women at risk of preterm birth for neuroprotection of the fetus. *Cochrane Database Syst. Rev.* 21:CD004661. 10.1002/14651858 17636771

[B26] Faulkner S. Bainbridge A. Kato T. Chandrasekaran M. Kapetanakis A. B. Hristova M. (2011). Xenon augmented hypothermia reduces early lactate/N-acetylaspartate and cell death in perinatal asphyxia. *Ann. Neurol.* 70 133–150. 10.1002/ana.22387 21674582

[B27] Forrest D. Yuzaki M. Soares H. D. Ng L. Luk D. C. Sheng M. (1994). Targeted disruption of NMDA receptor 1 gene abolishes NMDA response and results in neonatal death. *Neuron* 13 325–338. 10.1016/0896-6273(94)90350-6 8060614

[B28] Galinsky R. Bennet L. Groenendaal F. Lear C. A. Tan S. Van Bel F. (2014). Magnesium is not consistently neuroprotective for perinatal hypoxia-ischemia in term-equivalent models in preclinical studies: a systematic review. *Dev. Neurosci.* 36 73–82. 10.1159/000362206 24854050

[B29] Gerriets T. Stolz E. Walberer M. Kaps M. Bachmann G. Fisher M. (2003). Neuroprotective effects of MK-801 in different rat stroke models for permanent middle cerebral artery occlusion adverse: Effects of hypothalamic damage and strategies for its avoidance. *Stroke* 34 2234–2239. 10.1161/01.STR.0000087171.34637.A9 12920258

[B30] Glass H. C. (2018). Hypoxic-ischemic encephalopathy and other neonatal encephalopathies. *Contin. Lifelong Learn. Neurol.* 24 57–71. 10.1212/CON.0000000000000557 29432237

[B31] Groenendaal F. Rademaker C. M. Toet M. C. de Vries L. S. (2002). Effects of magnesium sulphate on amplitude-integrated continuous EEG in asphyxiated term neonates. *Acta Paediatr. Int. J. Paediatr.* 91 1073–1077. 10.1080/080352502760311575 12434893

[B32] Gurd J. W. Bissoon N. Beesley P. W. Nakazawa T. Yamamoto T. Vannucci S. J. (2002). Differential effects of hypoxia-ischemia on subunit expression and tyrosine phosphorylation of the NMDA receptor in 7- and 21-day-old rats. *J. Neurochem.* 82 848–856. 10.1046/j.1471-4159.2002.01026.x 12358790

[B33] Hagberg H. Mallard C. Ferriero D. M. Vannucci S. J. Levison S. W. Vexler Z. S. (2015). The role of inflammation in perinatal brain injury. *Nat. Rev. Neurol.* 11 192–208. 10.1038/nrneurol.2015.13 25686754 PMC4664161

[B34] Hardingham G. E. (2009). Coupling of the NMDA receptor to neuroprotective and neurodestructive events. *Biochem. Soc. Trans.* 37(Pt 6), 1147–1160. 10.1042/BST0371147 19909238 PMC2837198

[B35] Hardingham G. E. Fukunaga Y. Bading H. (2002). Extrasynaptic NMDARs oppose synaptic NMDARs by triggering CREB shut-off and cell death pathways. *Nat. Neurosci.* 5 405–441. 10.1038/nn835 11953750

[B36] Hetman M. Kharebava G. (2006). Survival Signaling Pathways Activated by NMDA Receptors. *Curr. Top. Med. Chem.* 6 787–799. 10.2174/156802606777057553 16719817

[B37] Hobbs C. Thoresen M. Tucker A. Aquilina K. Chakkarapani E. Dingley J. (2008). Xenon and hypothermia combine additively, offering long-term functional and histopathologic neuroprotection after neonatal hypoxia/ischemia. *Stroke* 39 1307–1313. 10.1161/STROKEAHA.107.499822 18309163

[B38] Hua C. Ju W. N. Jin H. Sun X. Zhao G. (2017). Molecular chaperones and hypoxic-ischemic encephalopathy. *Neural Regen. Res.* 12 153–160. 10.4103/1673-5374.199008 28250763 PMC5319223

[B39] Huang B. Y. Castillo M. (2008). Hypoxic-Ischemic brain injury: Imaging findings from birth to adulthood. *Radiographics* 28 417–439. 10.1148/rg.282075066 18349449

[B40] Ikonomidou C. Bosch F. Miksa M. Bittigau P. Vöckler J. Dikranian K. (1999). Blockade of NMDA receptors and apoptotic neurodegeneration in the developing brain. *Science* 283 70–74. 10.1126/science.283.5398.70 9872743

[B41] Impey S. Fong A. L. Wang Y. Cardinaux J. R. Fass D. M. Obrietan K. (2002). Phosphorylation of CBP mediates transcriptional activation by neural activity and CaM kinase IV. *Neuron* 34 235–244. 10.1016/S0896-6273(02)00654-2 11970865

[B42] Jacobs S. E. Morley C. J. Inder T. E. Stewart M. J. Smith K. R. McNamara P. J. (2011). Whole-body hypothermia for term and near-term newborns with hypoxic-ischemic encephalopathy: a randomized controlled trial. *Arch. Pediatr. Adolesc. Med.* 165 692–700. 10.1001/archpediatrics.2011.43 21464374

[B43] Janowska J. Gargas J. Ziemka-Nalecz M. Zalewska T. Sypecka J. (2020). Oligodendrocyte response to pathophysiological conditions triggered by episode of perinatal hypoxia-ischemia: role of IGF-1 secretion by glial cells. *Mol. Neurobiol.* 57 4250–4268. 10.1007/s12035-020-02015-z 32691304 PMC7467917

[B44] Jantzie L. L. Talos D. M. Jackson M. C. Park H. K. Graham D. A. Lechpammer M. (2015). Developmental expression of N-methyl-d-aspartate (n.d.) receptor subunits in human white and gray matter: Potential mechanism of increased vulnerability in the immature brain. *Cereb. Cortex* 57 4250–4268. 10.1093/cercor/bht246 24046081 PMC4303802

[B45] Jellema R. K. Lima Passos V. Zwanenburg A. Ophelders D. R. M. G. De Munter S. Vanderlocht J. (2013). Cerebral inflammation and mobilization of the peripheral immune system following global hypoxia-ischemia in preterm sheep. *J. Neuroinflamm.* 10:13. 10.1186/1742-2094-10-13 23347579 PMC3614445

[B46] Jevtovic-Todorovic V. Hartman R. E. Izumi Y. Benshoff N. D. Dikranian K. Zorumski C. F. (2003). Early exposure to common anesthetic agents causes widespread neurodegeneration in the developing rat brain and persistent learning deficits. *J. Neurosci.* 23 867–882. 10.1523/jneurosci.23-03-00876.2003 12574416 PMC6741934

[B47] Jiang X. Knox R. Pathipati P. Ferriero D. (2011). Developmental localization of NMDA receptors, Src and MAP kinases in mouse brain. *Neurosci. Lett.* 503 215–219. 10.1016/j.neulet.2011.08.039 21896318 PMC3193348

[B48] Jiang X. Mu D. Biran V. Faustino J. Chang S. Rincón C. M. (2008). Activated Src kinases interact with the N-methyl-D-aspartate receptor after neonatal brain ischemia. *Ann. Neurol.* 63 632–641. 10.1002/ana.21365 18384166

[B49] Johnston M. V. Nakajima W. Hagberg H. (2002). Mechanisms of hypoxic neurodegeneration in the developing brain. *Neuroscientist* 8 212–220. 10.1177/1073858402008003007 12061501

[B50] Johnston M. V. Trescher W. H. Ishida A. Nakajima W. Zipursky A. (2001). Neurobiology of hypoxic-ischemic injury in the developing brain. *Pediatr. Res.* 49 735–741. 10.1203/00006450-200106000-00003 11385130

[B51] Joyal J. L. Burks D. J. Pons S. Matter W. F. Vlahos C. J. White M. F. (1997). Calmodulin activates phosphatidylinositol 3-kinase. *J. Biol. Chem.* 272 28183–28186. 10.1074/jbc.272.45.28183 9353264

[B52] Juul S. E. Ferriero D. M. (2014). Pharmacologic neuroprotective strategies in neonatal brain injury. *Clin. Perinatol.* 41 119–131. 10.1016/j.clp.2013.09.004 24524450 PMC3929237

[B53] Káradóttir R. Cavelier P. Bergersen L. H. Attwell D. (2005). NMDA receptors are expressed in oligodendrocytes and activated in ischaemia. *Nature* 438 1162–1166. 10.1038/nature04302 16372011 PMC1416283

[B54] Kaur C. Rathnasamy G. Ling E. A. (2013). Roles of activated microglia in hypoxia induced neuroinflammation in the developing brain and the retina. *J. Neuroimmune Pharmacol.* 8 66–78. 10.1007/s11481-012-9347-2 22367679

[B55] Kim J. Y. Kim N. Yenari M. A. Chang W. (2011). Mild hypothermia suppresses calcium-sensing receptor (CaSR) induction following forebrain ischemia while increasing GABA-B receptor 1 (GABA-B-R1) Expression. *Transl. Stroke Res.* 2 195–201. 10.1007/s12975-011-0082-4 21731589 PMC3124781

[B56] Knox R. Brennan-Minnella A. M. Lu F. Yang D. Nakazawa T. Yamamoto T. (2014). NR2B phosphorylation at tyrosine 1472 contributes to brain injury in a rodent model of neonatal hypoxia-ischemia. *Stroke* 45 3040–3047. 10.1161/STROKEAHA.114.006170 25158771 PMC4175024

[B57] Knox R. Zhao C. Miguel-Perez D. Wang S. Yuan J. Ferriero D. (2013). Enhanced NMDA receptor tyrosine phosphorylation and increased brain injury following neonatal hypoxia-ischemia in mice with neuronal Fyn overexpression. *Neurobiol. Dis.* 51 113–119. 10.1016/j.nbd.2012.10.024 23127881 PMC3595007

[B58] Koehler R. C. Yang Z. J. Lee J. K. Martin L. J. (2018). Perinatal hypoxic-ischemic brain injury in large animal models: relevance to human neonatal encephalopathy. *J. Cereb. Blood Flow Metab.* 38 2092–2111. 10.1177/0271678X18797328 30149778 PMC6282216

[B59] Koh H. S. Chang C. Y. Jeon S. B. Yoon H. J. Ahn Y. H. Kim H. S. (2015). The HIF-1/glial TIM-3 axis controls inflammation-associated brain damage under hypoxia. *Nat. Commun.* 6:6340. 10.1038/ncomms7340 25790768 PMC4383004

[B60] Koodziejczyk K. Hamilton N. B. Wade A. Kradttir R. Attwell D. (2009). The effect of N-acetyl-aspartyl-glutamate and N-acetyl-aspartate on white matter oligodendrocytes. *Brain* 132(Pt 6), 1496–1508. 10.1093/brain/awp087 19383832 PMC2685922

[B61] Koziakova M. Harris K. Edge C. J. Franks N. P. White I. L. Dickinson R. (2019). Noble gas neuroprotection: xenon and argon protect against hypoxic–ischaemic injury in rat hippocampus in vitro via distinct mechanisms. *Br. J. Anaesth.* 123 601–609. 10.1016/j.bja.2019.07.010 31470983 PMC6871267

[B62] Lai Q. Hu P. Li Q. Li X. Yuan R. Tang X. (2016). NMDA receptors promote neurogenesis in the neonatal rat subventricular zone following hypoxic-ischemic injury. *Mol. Med. Rep.* 13 206–212. 10.3892/mmr.2015.4501 26548659 PMC4686072

[B63] Lai T. W. Shyu W. C. Wang Y. T. (2011). Stroke intervention pathways: NMDA receptors and beyond. *Trends Mol. Med.* 17 266–275. 10.1016/j.molmed.2010.12.008 21310659

[B64] Landucci E. Filippi L. Gerace E. Catarzi S. Guerrini R. Pellegrini-Giampietro D. E. (2018). Neuroprotective effects of topiramate and memantine in combination with hypothermia in hypoxic-ischemic brain injury in vitro and in vivo. *Neurosci. Lett.* 668 103–107. 10.1016/j.neulet.2018.01.023 29339173

[B65] Lapchak P. A. (2010). A critical assessment of edaravone acute ischemic stroke efficacy trials: Is edaravone an effective neuroprotective therapy? *Expert Opin. Pharmacother.* 11 1753–1763. 10.1517/14656566.2010.493558 20491547 PMC2891515

[B66] Lau C. G. Zukin R. S. (2007). NMDA receptor trafficking in synaptic plasticity and neuropsychiatric disorders. *Nat. Rev. Neurosci.* 8 413–426. 10.1038/nrn2153 17514195

[B67] Lee M. Y. Kuan Y. H. Chen H. Y. Chen T. Y. Chen S. T. Huang C. C. (2007). Intravenous administration of melatonin reduces the intracerebral cellular inflammatory response following transient focal cerebral ischemia in rats. *J. Pineal Res.* 42 297–309. 10.1111/j.1600-079X.2007.00420.x 17349029

[B68] Li B. Concepcion K. Meng X. Zhang L. (2017). Brain-immune interactions in perinatal hypoxic-ischemic brain injury. *Prog. Neurobiol.* 159 50–68. 10.1016/j.pneurobio.2017.10.006 29111451 PMC5831511

[B69] Lian X. Y. Stringer J. L. (2004). Astrocytes contribute to regulation of extracellular calcium and potassium in the rat cerebral cortex during spreading depression. *Brain Res.* 1012 177–184. 10.1016/j.brainres.2004.04.011 15158175

[B70] Lim I. A. Hall D. D. Hell J. W. (2002). Selectivity and promiscuity of the first and second PDZ domains of PSD-95 and synapse-associated protein 102. *J. Biol. Chem.* 277 21697–21711. 10.1074/jbc.M112339200 11937501

[B71] Liu C. W. Liao K. H. Tseng H. Wu C. M. Chen H. Y. Lai T. W. (2020). Hypothermia but not NMDA receptor antagonism protects against stroke induced by distal middle cerebral arterial occlusion in mice. *PLoS One* 15:e0229499. 10.1371/journal.pone.0229499 32126102 PMC7053748

[B72] Liu X. B. Murray K. D. Jones E. G. (2004). Switching of NMDA receptor 2A and 2B subunits at thalamic and cortical synapses during early postnatal development. *J. Neurosci.* 24 8885–8895. 10.1523/JNEUROSCI.2476-04.2004 15470155 PMC6729956

[B73] Liu Y. Wong T. P. Aarts M. Rooyakkers A. Liu L. Lai T. W. (2007). NMDA receptor subunits have differential roles in mediating excitotoxic neuronal death both in vitro and in vivo. *J. Neurosci.* 27 2846–2857. 10.1523/JNEUROSCI.0116-07.2007 17360906 PMC6672582

[B74] Lu F. Shao G. Wang Y. Guan S. Burlingame A. L. Liu X. (2018). Hypoxia-ischemia modifies postsynaptic GluN2B-containing NMDA receptor complexes in the neonatal mouse brain. *Exp. Neurol.* 299(Pt A), 65–74. 10.1016/j.expneurol.2017.10.005 28993251 PMC5723550

[B75] Lynch D. R. Guttmann R. P. (2002). Excitotoxicity: perspectives based on N-methyl-D-aspartate receptor subtypes. *J. Pharmacol. Exp. Ther.* 300 717–723. 10.1124/jpet.300.3.717 11861773

[B76] Ma D. Hossain M. Chow A. Arshad M. Battson R. M. Sanders R. D. (2005). Xenon and hypothermia combine to provide neuroprotection from neonatal asphyxia. *Ann. Neurol.* 58 182–193. 10.1002/ana.20547 16049939

[B77] Makarewicz D. Sulejczak D. Duszczyk M. Małek M. Słomka M. Lazarewicz J. W. (2014). Delayed preconditioning with NMDA receptor antagonists in a rat model of perinatal asphyxia. *Folia Neuropathol.* 52 270–284. 10.5114/fn.2014.45568 25310738

[B78] Mamun A. A. Yu H. Sharmeen R. McCullough L. D. Liu F. (2020). IRF5 signaling in phagocytes is detrimental to neonatal hypoxic ischemic encephalopathy. *Transl. Stroke. Res.* 10.1007/s12975-020-00832-x [Epub ahead of print]. 32761315 PMC7862420

[B79] Martel M. A. Wyllie D. J. A. Hardingham G. E. (2009). In developing hippocampal neurons, NR2B-containing N-methyl-d-aspartate receptors (NMDARs) can mediate signaling to neuronal survival and synaptic potentiation, as well as neuronal death. *Neuroscience* 158 334–343. 10.1016/j.neuroscience.2008.01.080 18378405 PMC2635533

[B80] Martinello K. Hart A. R. Yap S. Mitra S. Robertson N. J. (2017). Management and investigation of neonatal encephalopathy: 2017 update. *Arch. Dis. Child. Fetal Neonatal Edn.* 102 F346–F358. 10.1136/archdischild-2015-309639 28389438 PMC5537522

[B81] Matcovitch-Natan O. Winter D. R. Giladi A. Aguilar S. V. Spinrad A. Sarrazin S. (2016). Microglia development follows a stepwise program to regulate brain homeostasis. *Science* 353:aad8670. 10.1126/science.aad8670 27338705

[B82] Matute C. Domercq M. Sánchez-Gómez M. V. (2006). Glutamate-mediated glial injury: mechanisms and clinical importance. *Glia* 53 212–224. 10.1002/glia.20275 16206168

[B83] Maxwell J. R. Zimmerman A. J. Pavlik N. Newville J. C. Carlin K. Robinson S. (2020). Neonatal hypoxic-ischemic encephalopathy yields permanent deficits in learning acquisition: a preclinical touchscreen assessment. *Front. Pediatr.* 8:289. 10.3389/fped.2020.00289 32582593 PMC7291343

[B84] McDonald J. W. Silverstein F. S. Johnston M. V. (1987). MK-801 protects the neonatal brain from hypoxic-ischemic damage. *Eur. J. Pharmacol.* 140 359–361. 10.1016/0014-2999(87)90295-0 2820765

[B85] Merchant N. M. Azzopardi D. V. Hawwa A. F. Mcelnay J. C. Middleton B. Arendt J. (2013). Pharmacokinetics of melatonin in preterm infants. *Br. J. Clin. Pharmacol.* 76 725–733. 10.1111/bcp.12092 23432339 PMC3853531

[B86] Millar L. J. Shi L. Hoerder-Suabedissen A. Molnár Z. (2017). Neonatal hypoxia ischaemia: mechanisms, models, and therapeutic challenges. *Front. Cell Neurosci.* 11:78. 10.3389/fncel.2017.00078 28533743 PMC5420571

[B87] Miller J. T. Bartley J. H. Wimborne H. J. C. Walker A. L. Hess D. C. Hill W. D. (2005). The neuroblast and angioblast chemotaxic factor SDF-1 (CXCL12) expression is briefly up regulated by reactive astrocytes in brain following neonatal hypoxic-ischemic injury. *BMC Neurosci.* 6:63. 10.1186/1471-2202-6-63 16259636 PMC1298306

[B88] Monyer H. Burnashev N. Laurie D. J. Sakmann B. Seeburg P. H. (1994). Developmental and regional expression in the rat brain and functional properties of four NMDA receptors. *Neuron* 12 529–540. 10.1016/0896-6273(94)90210-0 7512349

[B89] Nakazawa T. Komai S. Tezuka T. Hisatsune C. Umemori H. Semba K. (2001). Characterization of Fyn-mediated tyrosine phosphorylation sites on GluRε2 (NR2B) subunit of the N-methyl-D-aspartate receptor. *J. Biol. Chem.* 276 693–699. 10.1074/jbc.M008085200 11024032

[B90] Niethammer M. Kim E. Sheng M. (1996). Interaction between the C terminus of NMDA receptor subunits and multiple members of the PSD-95 family of membrane-associated guanylate kinases. *J. Neurosci.* 16 2157–2163. 10.1523/jneurosci.16-07-02157.1996 8601796 PMC6578538

[B91] Nikizad H. Yon J. H. Carter L. B. Jevtovic-Todorovic V. (2007). Early exposure to general anesthesia causes significant neuronal deletion in the developing rat brain. *Ann. N. Y. Acad. Sci.* 1122 69–82. 10.1196/annals.1403.005 18077565

[B92] Nonomura M. Harada S. Asada Y. Matsumura H. Iwami H. Tanaka Y. (2019). Combination therapy with erythropoietin, magnesium sulfate and hypothermia for hypoxic-ischemic encephalopathy: An open-label pilot study to assess the safety and feasibility 11 medical and health sciences 1117 public health and health services. *BMC Pediatr.* 19:13. 10.1186/s12887-018-1389-z 30621649 PMC6325796

[B93] Odorcyk F. K. Duran-Carabali L. E. Rocha D. S. Sanches E. F. Martini A. P. Venturin G. T. (2020). Differential glucose and beta-hydroxybutyrate metabolism confers an intrinsic neuroprotection to the immature brain in a rat model of neonatal hypoxia ischemia. *Exp. Neurol.* 330:113317. 10.1016/j.expneurol.2020.113317 32304750

[B94] Olney J. W. Ikonomidou C. Mosinger J. L. Frierdich G. (1989). MK-801 prevents hypobaric-ischemic neuronal degeneration in infant rat brain. *J. Neurosci.* 9 1701–1704. 10.1523/jneurosci.09-05-01701.1989 2656934 PMC6569821

[B95] Olney J. W. Wozniak D. F. Jevtovic-Todorovic V. Farber N. B. Bittigau P. Ikonomidou C. (2002). Drug-induced apoptotic neurodegeneration in the developing brain. *Brain Pathol.* 12 488–498. 10.1111/j.1750-3639.2002.tb00467.x 12408236 PMC8095833

[B96] Oskoui M. Coutinho F. Dykeman J. Jetté N. Pringsheim T. (2013). An update on the prevalence of cerebral palsy: a systematic review and meta-analysis. *Dev. Med. Child Neurol.* 55 509–519. 10.1111/dmcn.12080 23346889

[B97] Paoletti P. Bellone C. Zhou Q. (2013). NMDA receptor subunit diversity: Impact on receptor properties, synaptic plasticity and disease. *Nat. Rev. Neurosci.* 14 383–400. 10.1038/nrn3504 23686171

[B98] Penrice J. Amess P. N. Punwani S. Wylezinska M. Tyszczuk L. D’Souza P. (1997). Magnesium sulfate after transient hypoxia-ischemia fails to prevent delayed cerebral energy failure in the newborn piglet. *Pediatr. Res.* 41 443–447. 10.1203/00006450-199703000-00024 9078550

[B99] Perlman J. M. (1997). Intrapartum hypoxic-ischemic cerebral injury and cerebral palsy: Medicolegal issues. *Pediatrics* 99 851–859. 10.1542/peds.99.6.851 9164779

[B100] Perlman J. M. (2006). Summary proceedings from the neurology group on hypoxic-ischemic encephalopathy. *Pediatrics* 117(3 Pt 2), S28–S33. 10.1542/peds.2005-0620E 16777819

[B101] Rahman S. Siham A. Tuzun H. Rahmani A. Rahman M. Rijims M. (2015). Multicenter randomized controlled trial of therapeutic hypothermia plus magnesium sulfate versus therapeutic hypothermia plus placebo in the management of term and near-term infants with hypoxic ischemic encephalopathy (The Mag Cool study): a pilot study. *J. Clin. Neonatol.* 4 158–163. 10.4103/2249-4847.159863

[B102] Rocha-Ferreira E. Hristova M. (2015). Antimicrobial peptides and complement in neonatal hypoxia-ischemia induced brain damage. *Front. Immunol.* 6:56. 10.3389/fimmu.2015.00056 25729383 PMC4325932

[B103] Rocha-Ferreira E. Hristova M. (2016). Plasticity in the neonatal brain following hypoxic-ischaemic injury. *Neural Plast.* 2016:4901014. 10.1155/2016/4901014 27047695 PMC4800097

[B104] Rosenberg P. A. Aizenman E. (1989). Hundred-fold increase in neuronal vulnerability to glutamate toxicity in astrocyte-poor cultures of rat cerebral cortex. *Neurosci. Lett.* 103 162–168. 10.1016/0304-3940(89)90569-7 2570387

[B105] Rosenberg P. A. Amin S. Leitner M. (1992). Glutamate uptake disguises neurotoxic potency of glutamate agonists in cerebral cortex in dissociated cell culture. *J. Neurosci.* 12 56–61. 10.1523/jneurosci.12-01-00056.1992 1345946 PMC6575706

[B106] Rüegger C. M. Davis P. G. Cheong J. L. (2017). Xenon as an adjuvant to therapeutic hypothermia in near term and term newborns with hypoxic ischaemic encephalopathy. *Cochrane Database Syst. Rev.* 8:CD012753. 10.1002/14651858 30123976 PMC6513612

[B107] Rüegger C. M. Davis P. G. Cheong J. L. (2018). Xenon as an adjuvant to therapeutic hypothermia in near-term and term newborns with hypoxic-ischaemic encephalopathy. *Cochrane Database Syst. Rev.* 8:CD0 12753. 10.1002/14651858.CD012753.pub2 PMC6513612 30123976

[B108] Salter M. G. Fern R. (2005). NMDA receptors are expressed in developing oligodendrocyte processes and mediate injury. *Nature* 438 1167–1171. 10.1038/nature04301 16372012

[B109] Sánchez-Gómez M. V. Alberdi E. Ibarretxe G. Torre I. Matute C. (2003). Caspase-dependent and caspase-independent oligodendrocyte death mediated by AMPA and kainate receptors. *J. Neurosci.* 23 9519–9528. 10.1523/jneurosci.23-29-09519.2003 14573531 PMC6740470

[B110] Shao G. Wang Y. Guan S. Burlingame A. L. Lu F. Knox R. (2017). Proteomic analysis of mouse cortex postsynaptic density following neonatal brain hypoxia-ischemia. *Dev. Neurosci.* 39 66–81. 10.1159/000456030 28315865 PMC5519436

[B111] Shea K. L. Palanisamy A. (2015). What can you do to protect the newborn brain? *Curr. Opin. Anaesthesiol.* 28 261–266. 10.1097/ACO.0000000000000184 25827279

[B112] Sheng M. Cummings J. Roldan L. A. Jan Y. N. Jan L. Y. (1994). Changing subunit composition of heteromeric NMDA receptors during development of rat cortex. *Nature* 368 144–147. 10.1038/368144a0 8139656

[B113] Solevåg A. L. Schmölzer G. M. Cheung P. Y. (2019). Novel interventions to reduce oxidative-stress related brain injury in neonatal asphyxia. *Free Radic. Biol. Med.* 142 113–122. 10.1016/j.freeradbiomed.2019.04.028 31039399

[B114] Soriano F. X. Martel M. A. Papadia S. Vaslin A. Baxter P. Rickman C. (2008). Specific targeting of pro-death NMDA receptor signals with differing reliance on the NR2B PDZ ligand. *J. Neurosci.* 28 10696–10710. 10.1523/JNEUROSCI.1207-08.2008 18923045 PMC2602846

[B115] Sun H. S. Doucette T. A. Liu Y. Fang Y. Teves L. Aarts M. (2008). Effectiveness of PSD95 inhibitors in permanent and transient focal ischemia in the rat. *Stroke* 39 2544–2553. 10.1161/STROKEAHA.107.506048 18617669

[B116] Sun Y. Zhang L. Chen Y. Zhan L. Gao Z. (2015). Therapeutic targets for cerebral ischemia based on the signaling pathways of the GluN2B C terminus. *Stroke* 46 2347–2353. 10.1161/STROKEAHA.115.009314 26173725

[B117] Sun Y. J. Zhang Z. Y. Fan B. Li G. Y. (2019). Neuroprotection by therapeutic hypothermia. *Front. Neurosci.* 13:584. 10.3389/fnins.2019.00586 31244597 PMC6579927

[B118] Szydlowska K. Tymianski M. (2010). Calcium, ischemia and excitotoxicity. *Cell Calcium* 47 122–129. 10.1016/j.ceca.2010.01.003 20167368

[B119] Tagin M. A. Woolcott C. G. Vincer M. J. Whyte R. K. Stinson D. A. (2012). Hypothermia for neonatal hypoxic ischemic encephalopathy: an updated systematic review and meta-analysis. *Arch. Pediatr. Adolesc. Med.* 166 558–566. 10.1001/archpediatrics.2011.1772 22312166

[B120] Takagi N. Sasakawa K. Besshoh S. Miyake-Takagi K. Takeo S. (2003). Transient ischemia enhances tyrosine phosphorylation and binding of the NMDA receptor to the Src homology 2 domain of phosphatidylinositol 3-kinase in the rat hippocampus. *J. Neurochem.* 84 67–76. 10.1046/j.1471-4159.2003.01500.x 12485402

[B121] Takagi N. Shinno K. Teves L. Bissoon N. Wallace M. C. Gurd J. W. (2002). Transient ischemia differentially increases tyrosine phosphorylation of NMDA receptor subunits 2A and 2B. *J. Neurochem.* 69 1060–1065. 10.1046/j.1471-4159.1997.69031060.x 9282928

[B122] Terasaki Y. Sasaki T. Yagita Y. Okazaki S. Sugiyama Y. Oyama N. (2010). Activation of NR2A receptors induces ischemic tolerance through CREB signaling. *J. Cereb. Blood Flow Metab.* 30 1441–1449. 10.1038/jcbfm.2010.18 20145658 PMC2949236

[B123] Thoresen M. Penrice J. Lorek A. Cady E. B. Wylezinska M. Kirkbride V. (1995). Mild hypothermia after severe transient hypoxia-ischemia ameliorates delayed cerebral energy failure in the newborn piglet. *Pediatr. Res.* 37 667–670. 10.1203/00006450-199505000-00019 7603788

[B124] Toriuchi K. Kakita H. Tamura T. Takeshita S. Yamada Y. Aoyama M. (2020). Prolonged astrocyte-derived erythropoietin expression attenuates neuronal damage under hypothermic conditions. *J. Neuroinflamm.* 17:141. 10.1186/s12974-020-01831-3 32359362 PMC7195727

[B125] Traynelis S. F. Wollmuth L. P. McBain C. J. Menniti F. S. Vance K. M. Ogden K. K. (2010). Glutamate receptor ion channels: Structure, regulation, and function. *Pharmacol. Rev.* 62 405–496. 10.1124/pr.109.002451 20716669 PMC2964903

[B126] Trotman M. Vermehren P. Gibson C. L. Fern R. (2015). The dichotomy of memantine treatment for ischemic stroke: dose-dependent protective and detrimental effects. *J. Cereb. Blood Flow Metab.* 35 230–239. 10.1038/jcbfm.2014.188 25407270 PMC4426739

[B127] Tu W. Xu X. Peng L. Zhong X. Zhang W. Soundarapandian M. M. (2010). DAPK1 interaction with NMDA receptor NR2B subunits mediates brain damage in stroke. *Cell* 140 222–234. 10.1016/j.cell.2009.12.055 20141836 PMC2820131

[B128] van Zundert B. Yoshii A. Constantine-Paton M. (2004). Receptor compartmentalization and trafficking at glutamate synapses: a developmental proposal. *Trends Neurosci.* 27 428–437. 10.1016/j.tins.2004.05.010 15219743

[B129] Vanhoutte P. Bading H. (2003). Opposing roles of synaptic and extrasynaptic NMDA receptors in neuronal calcium signalling and BDNF gene regulation. *Curr. Opin. Neurobiol.* 13 366–371. 10.1016/S0959-4388(03)00073-4 12850222

[B130] Wassink G. Davidson J. O. Dhillon S. K. Zhou K. Bennet L. Thoresen M. (2019). Therapeutic hypothermia in neonatal hypoxic-ischemic encephalopathy. *Curr. Neurol. Neurosci. Rep.* 19:2. 10.1007/s11910-019-0916-0 30637551

[B131] Wassink G. Davidson J. O. Fraser M. Yuill C. A. Bennet L. Gunn A. J. (2020). Non-additive effects of adjunct erythropoietin therapy with therapeutic hypothermia after global cerebral ischaemia in near-term fetal sheep. *J. Physiol.* 598 999–1015. 10.1113/JP279131 31912503

[B132] Wassink G. Gunn E. R. Drury P. P. Bennet L. Gunn A. J. (2014). The mechanisms and treatment of asphyxial encephalopathy. *Front. Neurosci.* 8:40. 10.3389/fnins.2014.00040 24578682 PMC3936504

[B133] Wu G. Y. Deisseroth K. Tsien R. W. (2001). Activity-dependent CREB phosphorylation: Convergence of a fast, sensitive calmodulin kinase pathway and a slow, less sensitive mitogen-activated protein kinase pathway. *Proc. Natl. Acad. Sci. U.S.A.* 98 2808–2813. 10.1073/pnas.051634198 11226322 PMC30221

[B134] Wu Q. J. Tymianski M. (2018). Targeting nmda receptors in stroke: new hope in neuroprotection. *Mol. Brain.* 11:15. 10.1186/s13041-018-0357-8 29534733 PMC5851248

[B135] Wu Y. Chen C. Yang Q. Jiao M. Qiu S. (2017). Endocytosis of GluN2B-containing NMDA receptor mediates NMDA-induced excitotoxicity. *Mol. Pain* 13:1744806917701921. 10.1177/1744806917701921 28326942 PMC5391130

[B136] Wu Y. W. Bauer L. A. Ballard R. A. Ferriero D. M. Glidden D. V. Mayock D. E. (2012). Erythropoietin for neuroprotection in neonatal encephalopathy: Safety and pharmacokinetics. *Pediatrics* 130 683–691. 10.1542/peds.2012-0498 23008465 PMC3457622

[B137] Yang S. N. Lai M. C. (2011). Perinatal hypoxic-ischemic encephalopathy. *J. Biomed. Biotechnol.* 2011:609813. 10.1155/2011/609813 21197402 PMC3010686

[B138] Zhang S. J. Steijaert M. N. Lau D. Schütz G. Delucinge-Vivier C. Descombes P. (2007). Decoding NMDA receptor signaling: identification of genomic programs specifying neuronal survival and death. *Neuron* 53 549–562. 10.1016/j.neuron.2007.01.025 17296556

[B139] Zhao H. Mitchell S. Ciechanowicz S. Savage S. Wang T. Ji X. (2013). Argon combined with hypothermia protects against hypoxia-ischaemia-induced brain damage in rat neonates. *Br. J. Anaesth.* 125 180–192. 10.1093/bja/aes490

[B140] Zhao M. Zhu P. Fujino M. Zhuang J. Guo H. Sheikh I. (2016). Oxidative stress in hypoxic-ischemic encephalopathy: molecular mechanisms and therapeutic strategies. *Int. J. Mol. Sci.* 17:2078. 10.3390/ijms17122078 27973415 PMC5187878

[B141] Zhou K. Q. Davidson J. O. Bennet L. Gunn A. J. (2020). Combination treatments with therapeutic hypothermia for hypoxic-ischemic neuroprotection. *Dev. Med. Child Neurol.* 62 1131–1137. 10.1111/dmcn.14610 32614467

[B142] Zhou X. Ding Q. Chen Z. Yun H. Wang H. (2013). Involvement of the GluN2A and GluN2B subunits in synaptic and extrasynaptic N-methyl-D-aspartate receptor function and neuronal excitotoxicity. *J. Biol. Chem.* 288 24151–24159. 10.1074/jbc.M113.482000 23839940 PMC3745357

[B143] Zhu C. Kang W. Xu F. Cheng X. Zhang Z. Jia L. (2009). Erythropoietin improved neurologic outcomes in newborns with hypoxic-ischemic encephalopathy. *Pediatrics* 124 e218–e226. 10.1542/peds.2008-3553 19651565

[B144] Zhu H. D. Martin R. Meloni B. Oltvolgyi C. Moore S. Majda B. (2004). Magnesium sulfate fails to reduce infarct volume following transient focal cerebral ischemia in rats. *Neurosci. Res.* 49 347–353. 10.1016/j.neures.2004.04.001 15196783

[B145] Zhuang L. Yang T. Vizcaychipi M. P. Yu B. Ma D. (2010). Argon provides neuroprotection against ischemia/hypoxia induced brain damage in rat neonates. *Intensive Care Med.* 7 25640–25651. 10.1007/s00134-010-2001-7

[B146] Ziemka-Nalecz M. Janowska J. Strojek L. Jaworska J. Zalewska T. Frontczak-Baniewicz M. (2018). Impact of neonatal hypoxia-ischaemia on oligodendrocyte survival, maturation and myelinating potential. *J. Cell. Mol. Med.* 22 207–222. 10.1111/jcmm.13309 28782169 PMC5742723

